# The emerging role of ASC in dendritic cell metabolism during *Chlamydia* infection

**DOI:** 10.1371/journal.pone.0188643

**Published:** 2017-12-07

**Authors:** Danielle N. McKeithen, Yusuf O. Omosun, Khamia Ryans, Jing Mu, Zhonglin Xie, Tankya Simoneaux, Uriel Blas-machado, Francis O. Eko, Carolyn M. Black, Joseph U. Igietseme, Qing He

**Affiliations:** 1 Department Microbiology, Biochemistry, and, Immunology, Morehouse School of Medicine, Atlanta, GA, United States of America; 2 Department of Biology, Clark Atlanta University, Atlanta, GA, United States of America; 3 Center for Molecular and Translational Medicine, Georgia State University, Atlanta, GA, United States of America; 4 College of Veterinary Medicine, University of Georgia, Georgia, Atlanta, United States of America; 5 National Center for Emerging Zoonotic and Infectious Diseases, Centers for Disease Control & Prevention (CDC), Atlanta, GA, United States of America; University of the Pacific, UNITED STATES

## Abstract

*Chlamydia trachomatis* is a bacterial agent that causes sexually transmitted infections worldwide. The regulatory functions of dendritic cells (DCs) play a major role in protective immunity against Chlamydia infections. Here, we investigated the role of ASC in DCs metabolism and the regulation of DCs activation and function during *Chlamydia* infection. Following *Chlamydia* stimulation, maturation and antigen presenting functions were impaired in ASC^-/-^ DCs compared to wild type (WT) DCs, in addition, ASC deficiency induced a tolerant phenotype in *Chlamydia* stimulated DCs. Using real-time extracellular flux analysis, we showed that activation *in Chlamydia* stimulated WT DCs is associated with a metabolic change in which mitochondrial oxidative phosphorylation (OXPHOS) is inhibited and the cells become committed to utilizing glucose through aerobic glycolysis for differentiation and antigen presenting functions. However, in ASC^-/-^ DCs *Chlamydia*-induced metabolic change was prevented and there was a significant effect on mitochondrial morphology. The mitochondria of *Chlamydia* stimulated ASC^-/-^ DCs had disrupted cristae compared to the normal narrow pleomorphic cristae found in stimulated WT DCs. In conclusion, our results suggest that *Chlamydia*-mediated activation of DCs is associated with a metabolic transition in which OXPHOS is inhibited, thereby dedicating the DCs to aerobic glycolysis, while ASC deficiency disrupts DCs function by inhibiting the reprogramming of DCs metabolism within the mitochondria, from glycolysis to electron transport chain.

## Introduction

*Chlamydia trachomatis (C*. *trachomatis)* is an obligate intercellular Gram-negative bacterium that infects both humans and animal species. *Chlamydia* infections are the most common bacterial sexually transmitted infection (STI) within the US [1]. Known as the silent infection, the Chlamydial infection can persist within a host resulting in devastating damage of the reproductive system, leading to severe complications, which includes pelvic inflammatory disease (PID), ectopic pregnancy and tubal factor infertility (TFI) [2, 3–6].

This study hypothesis was derived from our previous study on the ability of IL10^-/-^ mice to have shorter duration of *Chlamydia* infection and reduced bacterial burden which we have attributed to the dendritic cells (DCs) being able to rapidly and preferentially activate a high Th1 response [7]. Inflammasomes are emerging as cytosolic defenses against infections; they not only detect major bacterial molecules, but also can sense bacterial virulence activities. Inflammasome activation modulates *Chlamydia* growth and infection through the production of proinflammatory cytokines [8–10]. A well-known inflammasome is the NLRP3 inflammasome, which has a representative design consisting of NLR, apoptosis-associated speck-like protein containing a CARD (ASC) adaptor protein, and caspase-1. ASC forms specks by self-oligomerization to activate caspase-1 and induce pyroptosis, a mechanism mediated by caspase-1 activation [11–13]. The effects of ASC on immune signaling are primarily through bridging the interaction between NLR proteins with caspase-1 to control the processing of the proinflammatory cytokines IL-1β and IL-18, ultimately determining the balance between pathogen resolution and disease pathology [14–16]. Animal experiments have clearly demonstrated that NLRP3 inflammasome is crucial for host defense against *C*. *pneumoniae* infection [17–20] and that ASC deficiency significantly increases bacterial colonization and delays genital *C*. *muridarum* infection clearance [21–23]. Monocytes and dendritic cells secrete IL-1β through the activation of inflammasomes during *C*. *trachomatis* infection [21, 23–25]. Evidence is emerging that point to the important role of ASC in controlling immune responses, for instance, adjuvanticity of the oil in-water emulsion MF59 was recently shown to require ASC to induce antigen-specific antibodies against influenza [26]. Moreover, granuloma formation and host defense in chronic *Mycobacterium tuberculosis* infection is ASC dependent [27]. Recent studies have pointed to the existence of cell-intrinsic roles for ASC in dendritic cell and lymphocyte populations [16]. We have recently reported that IL-10 regulates the expression of ASC and affected NLRP3 inflammasome activation by inhibiting its assembly in *Chlamydia* exposed DCs [25]. These scientific premises indicate that the ASC is involved in *Chlamydia* immunity and there is a need to further investigate the possible mechanisms by which ASC regulates host immunity during *Chlamydia* infection.

Over the past years, it has become increasingly clear that different stages of immune cell activation coincide with, and are underpinned by, different types of cellular metabolism that are tailored towards the bioenergetic and biosynthetic needs of these cells. Emerging data demonstrates the contribution of cellular metabolic pathways to the ability of immune cells to sense their microenvironment and alter their function [28, 29] [30]. Distinct alterations in the microenvironment induce metabolic programs that might form the basis of the inducible and reversible spectrum of functional immune cell activation/polarization phenotypes. For example, after exposure to Toll-like receptor agonists, DCs undergo a metabolic transition from oxidative phosphorylation (OXPHOS) to aerobic glycolysis, which supports DC maturation [28]. This alteration in metabolism is required to meet the increased biosynthetic and bioenergetic demands of activated DCs specifically by funneling metabolites into pathways for lipid and protein synthesis [31–33]. An exciting theme emerging from the recent reports is that inflammation and metabolism are more intimately linked than previously thought, with inflammasomes modulating carbohydrate, lipid metabolism, and the glycolysis pathway.

In this study, we have investigated aspects of the novel functional relationship between dendritic cell metabolic pathways and ASC during *Chlamydia* infection. The findings from this study will highlight possible means of regulating immune cell function, as well as providing possible immunotherapeutic strategies for inducing adequate and long-term immunity against *Chlamydia* infection, a disease of public health importance.

## Material and methods

### Mice

Six-week old female wild type (WT) mice with C57BL/6J background were purchased from The Jackson Laboratory and female ASC^-/-^ mice with a C57BL/6J background (provided by Dr. Dixit; Genentech). Food and water were provided *ad libitum* to the mice, which were kept in laminar-flow racks under pathogen-free conditions, with a 12-hr light and a 12-hr dark cycle. Mice were cared for by the Morehouse School of Medicine Research Animal Resources staff under a protocol approved by the Institutional Animal Care and Use Committee (MSM-IACUC) in accordance with specifications detailed in the Institute for Laboratory Animal Research (ILAR) Guide for the Care and Use of Laboratory Animals.

### *Chlamydia* stocks

Stocks of *Chlamydia muridarum* (*C*. *muridarum*), the agent of mouse pneumonitis, (Division of Scientific Research, Centers for Disease Control and Prevention) used for infections were prepared by propagating elementary bodies (EBs) in McCoy or HeLa cells, per standard procedures. The cell lines were maintained in Minimum Essential Medium (MEM) supplemented with Earle’s with 2mM L-glutamine, 10% heat-inactivated FBS, 1mM sodium pyruvate, 0.5% fungizone, and, 1.0% penicillin/streptomycin (100U/mL; 100μg/mL) in a humidified atmosphere under 5% CO2, at 37°C. Chlamydial stock titers were expressed as inclusion forming units (IFU) per milliliter.

### Infectivity

The WT and ASC^-/-^ mice (n = 8 per group) were anesthetized with sodium pentobarbital (30μg/body weight) (Sigma-Aldrich) and infected intravaginally with 20μl of 1 x 10^5^ IFUs of *C*. *muridarum* approximately 5 days after subcutaneous administration of 2.5 mg/mouse Depo Provera (Pfizer Inc.). Following infection, the mice were swabbed vaginally every three days for two weeks and once a week for the last two weeks. The course of the infection was monitored by isolation of *Chlamydia* from the cervicovaginal swabs cultured in 24 well plates with McCoy cells (Division of Scientific Research, Centers for Disease Control and Prevention) at 37°C, 5% CO_2_ for 1hr. Culture media was replaced with MEM media supplemented with 2mM L-glutamine, 10% heat-inactivated FBS, 1 mM sodium pyruvate, and, 0.5% fungizone and incubated at 37°C, 5%CO_2_ for an additional 48hrs. The Pathfinder, Chlamydia Culture Confirmation System (Bio-Rad) manufacture’s protocol was followed for immunofluorescence staining and IFU counting. The experiment was repeated three times.

### Bone marrow DC (BMDC) extraction

Immature DCs were isolated from the bone marrow of WT and ASC^-/-^ mice (n = 5 per group) by the standard method and differentiated *in vitro* by culturing with IL–4 and granulocyte-macrophage colony-stimulating factor (GM-CSF), as previously described (35). Briefly, bone marrow progenitor cells from mice femurs were removed, washed three times with RPMI 1640 supplemented with 10% FBS, and incubated in RPMI 1640 supplemented with 10% fetal bovine serum (FBS), 10 mM HEPES, 200 mM glutamine, 100 mM sodium pyruvate, 0.5% HEPES, 5ng/mL IL–4, and, 10ng/mL GM–CSF at 37C, replacing with fresh media on day 3 and transferring cells to new dishes on day 5. After 5 days in culture, the cells were characterized as loosely adherent mononuclear cells and further purified as CD11c expressing DCs by using the Pan Dendritic Cell Isolation Kit from Miltenyi Biotec.

### Adoptive transfer

Isolated DCs from WT and ASC^-/-^ mice were stimulated with *C*. *muridarum* EBs for 2hrs and adoptively transferred into 6–8 weeks old female WT mice (2.5 x 10^7^ cells/mouse) by intravenous infection into the tail vein in 0.2 mL of 1X phosphate-buffered saline (PBS) (n = 4 per group). Mice were infected intravaginally with 1 x 10^5^ IFU/mouse of live *C*. *muridarum* 1 week after adoptive transfer. The status of the infection was monitored by periodic cervicovaginal swabbing of individual animal and isolation of *Chlamydia* in tissue culture as previously described. The experiment was repeated three times.

### Flow cytometry

Single DCs suspensions from WT and ASC^-/-^ mice were infected at a ratio of 1:5 with *C*. *muridarum* for 2 hours at 37°C and washed with fluorescence-activated cell sorting (FACS) buffer at 4°C. Cells were stained with fluorescein isothiocyanate (FITC)-, phycoerythrin (PE)-, or allophycocyanin (APC)-conjugated antibodies against surface markers CD80, CD103, MHC II, CD40, CD14, TLR4, and CD11c (BD Pharmingen). Staining was performed in the presence of 5 μg/mL of F_c_ Blocker. Cell acquisition was performed on a guava EasyCyte 8HT. For each sample, at least 100,000 events were collected. Data was analyzed with GuavaSoft 2.7.

### Cytokine expression

Cultured supernatants from WT and ASC^-/-^ DCs were collected and analyzed by Bio-Plex Pro Mouse Cytokine 23-Plex multiplex array according to manufacturer’s guidelines (Bio-Rad) using a Luminex machine. The concentration of cytokine in each sample was obtained by extrapolation from a standard calibration curve generated simultaneously. The mean and SD of all replicate cultures were calculated. The experiment was repeated three times.

### Immunoblotting

Lysates from *Chlamydia* pulsed WT and ASC^-/-^ DCs were prepared by homogenization in lysis buffer supplemented with 1 mmol/L phenylmethylsulfonyl fluoride and protease inhibitor cocktail. 20μg protein of WT and ASC^-/-^ DC lysates and supernatants from cell cultures were loaded onto 4–20% TGX gradient gel (Bio-Rad) and run for 1 hour. Proteins were then transferred onto nitrocellulose paper (Bio-Rad). After 1 hour, the blots were washed, blocked with 5% milk, and then incubated with desired primary antibody overnight at 4°C. Horseradish peroxidase (HRP)-conjugated secondary antibodies (R&D Systems) were added for 1 hour at room temperature, and then the blots were developed using Clarity Western enhanced chemiluminescence (ECL) substrate (Bio-Rad). Viewing and quantification was analyzed using ImageQuant LAS 4000 (GE Healthcare). The mean and SD of all replicate cultures were calculated. The experiment was repeated three times.

### *In vitro* DC antigen presentation assays

Lymphocytes were obtained from spleens of *Chlamydia* infected WT and ASC^-/-^ mice using a 40μm filter and syringe plunger and suspended in PBS solution. The CD4^+^ T cells were then purified using the MACS mouse Pan T Cell Isolation Kit (mouse) (Miltenyi Biotec). To assess the antigen-presenting function of DCs from either ASC^-/-^ or WT mice, 1 x 10^5^ γ-irradiated DCs were co-cultured with 2 x 10^5^ purified T cells in the presence or absence of UV-inactivated *C*. *muridarum* in 96-well tissue culture plates for 5 days. The amounts of IL-2, -5, -9, -12, -13, -17A, IFN-γ, MIP-1β, RANTES, and TNF-α in the culture supernatants were measured using the Bio-Plex Pro Mouse Cytokine 23-Plex multiplex array per the manufacturer’s guidelines (Bio-Rad) using a Luminex machine. The concentration of cytokine in each sample was obtained by extrapolation from a standard calibration curve generated simultaneously. The mean and SD of all replicate cultures were calculated. The experiment was repeated two times. Antigen specific T cell proliferative response was assessed for their ability to proliferate in response to *in vitro* re-stimulation in culture with *C*. *muridarum*. Results are expressed as cell viability ratio which is ratio between the absorbance values of stimulated and non-stimulated cells and the bars represent the mean and SD of three independent experiments. The T cell proliferation was detected using Cell Signaling XTT Cell Viability Kit protocol.

### Metabolic analysis

Real-time changes in extracellular acidification rates (ECARs) and oxygen consumption rates (OCRs) were analyzed using extracellular flux analysis (Agilent Technology, Santa Clara, CA). In brief, DCs were plated in XFe-96 cell culture plates (2 × 10^5^ cells/well in 200 μl) and either left unstimulated or stimulated (*C*. *muridarum*) with indicated conditions. At indicated time points (20, 40, 60, 80 and 100 minutes), DCs were washed and analyzed in XF running buffer (unbuffered RPMI 1640, 10 mM glucose, 10% FCS, 100 U/ml penicillin/streptomycin, 2 mM l-glutamine, and 20 ng/ml GMCSF) per the manufacturer's instructions to obtain real-time measurements of OCRs and ECARs. Where indicated, ECARs and/or OCRs were analyzed in response to 1 μM oligomycin, 1.5 μM fluoro-carbonyl cyanide phenylhydrazone, and 100 nM rotenone plus 1 μM antimycin A (all from Sigma-Aldrich), or 500 μM SEITU as indicated. For pyruvate production assay, the WT and ASC^-/-^ DCs were stimulated with *C*. *muridarum* for 1 and 2 hours respectively. DCs supernatants were collected and analyzed for pyruvate using the Eton Bioscience Pyruvate Assay Kit. Each sample was incubated with the Pyruvate Assay Kit reaction solution in a 96-well flat bottom plate for 30 minutes at 37°C and measured at 570 nm. Included in the analysis were uninfected WT and ASC^-/-^ DCs and culture media, as control groups. The concentration of pyruvate from each sample was obtained by extrapolation from a standard calibration curve generated simultaneously. The mean and SD of all replicate cultures were calculated. The experiments were repeated three times.

### Transmission electron microscopy (TEM)

The WT and ASC^-/-^ DCs were infected with *C*. *muridarum* at a ratio of 1:5 for 2 hours in 24-well tissue culture plates. The cells were washed and fixed on a glass slide with 2.5% (w/v) glutaraldehyde/0.1 M cacodylate buffer for 2 to 6 hours at room temperature. After fixation, DCs were mounted, cut into 200 mm slices with a vibratome (EM Corp.), and post fixed in aqueous osmium tetroxide. Fixed DC slices were dehydrated using graded ethanol and propylene oxide, and embedded in Polybed 812 resin (Polysciences, Inc.). Thin sections (80 nm) were then cut with a diamond knife and stained with 5% uranyl acetate and Reynold’s lead citrate. A JEOL 1200EX transmission electron microscope was used to examine mitochondrial changes. The experiment was repeated three times.

### Statistical analysis

During this study, we were interested in determining the difference in response between WT and ASC^-/-^ DCs. Statistical analysis was used to compare Chlamydia infected WT mice with Chlamydia infected ASC^-/-^ mice and Chlamydia pulsed WT DCs with Chlamydia pulsed ASC^-/-^ DCs. The data derived from infectivity assay, cytokine analysis of DC supernatants, flow cytometric differentiation of DCs, cytokine analysis of co-culture supernatants, T cell proliferation assays and ELISA (Pyruvate concentration determination) were compared by performing a Student t-test. Differences were considered statistically significance when judged to have a P-value less than 0.05. Software packages used included Graphpad Prism, Excel and Sigmaplot.

## Results

### ASC deficiency results in higher number of IFUs following an acute *Chlamydia* infection

**Fig 1:**
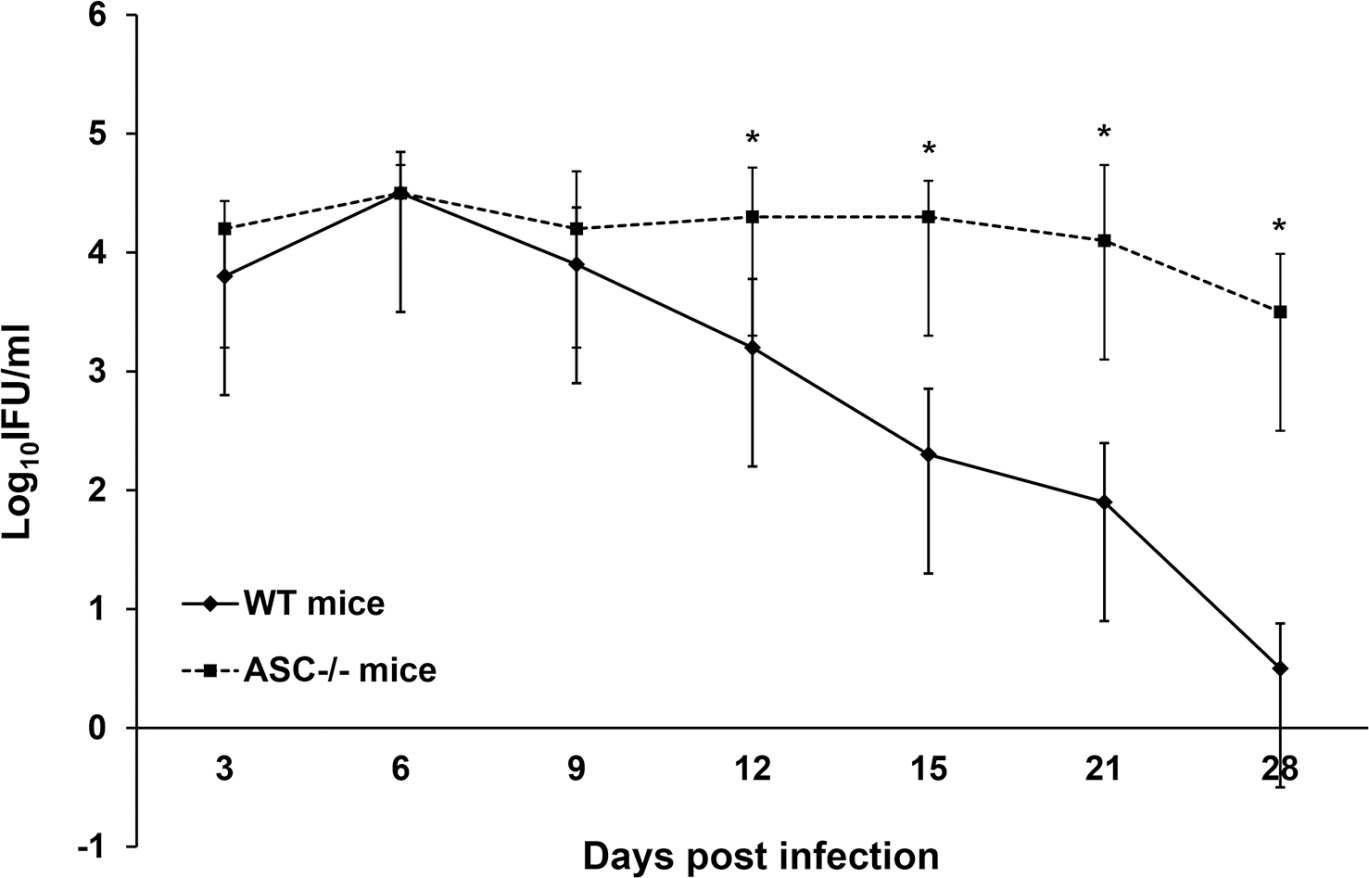
ASC deficiency results in higher number of IFUs following an acute *Chlamydia* infection. The WT and ASC^-/-^ mice were infected with *C*. *muridarum* and swabbed intravaginally every three days for two weeks and once a week for the last two weeks. *C*. *muridarum* IFUs was determined to monitor the course of the infection. Each data point depicts the mean ± SD of the individual numbers of recoverable IFUs from each mouse per group of 8 mice, at the indicated days post infection; expressed as Log_10_ IFU/ml ± SD. The results showed that *C*. *muridarum* infection took a longer time to clear in ASC^-/-^ mice. The experiment was repeated three times.

We first wanted to find an association between ASC deficiency and acute chlamydial infection. This was accomplished by comparing the course of genital chlamydial infection in WT and ASC^-/-^ mice. Our data showed that in WT mice the infection was nearly cleared by day 28 of the initial infection (Fig 1). Conversely, the numbers of chlamydial IFUs within ASC^-/-^ mice were significantly higher (p<0.05) from Day 15, and remained significantly high through to Day 28 in comparison to the WT mice. This data is lines up with other reports [20].

### Expression of proteins associated with the inflammasome

**Fig 2:**
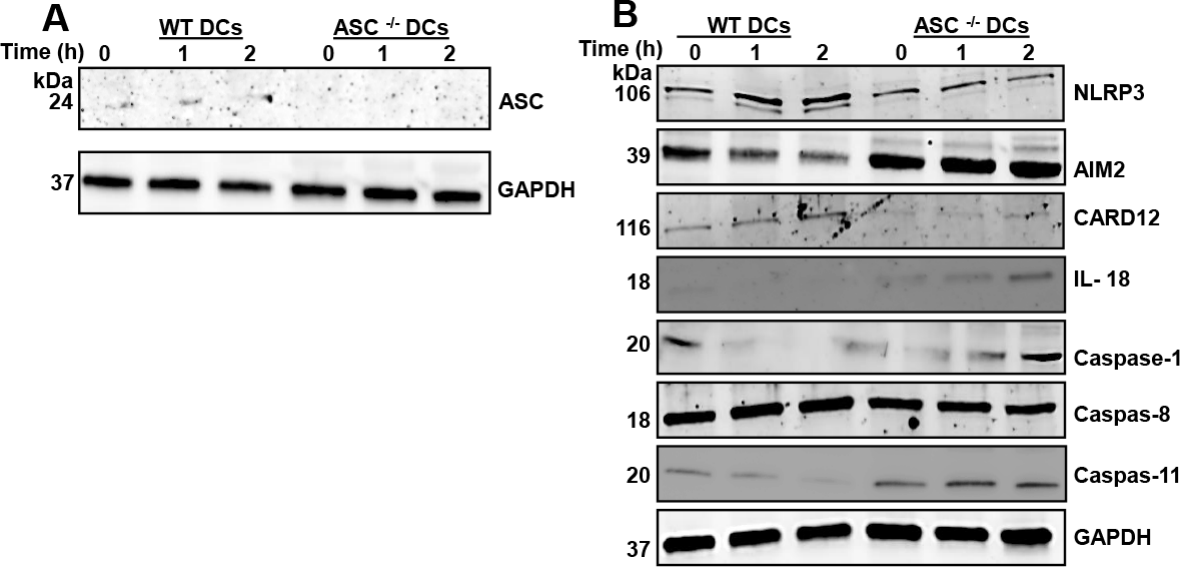
Expression of proteins associated with the inflammasome. Representative Western blotting was used to probe for A). ASC in lysates from WT and ASC^-/-^ DCs that were stimulated *with C*. *muridarum* for 0, 1 and 2 hours after infection. We show that the expression of ASC was completely abolished in ASC^-/-^ DCs. B). NLRP3, AIM2, CARD 12, IL-18, Caspase-1, Caspase-8 and Caspase-11 expression in lysates from WT and ASC^-/-^ DCs that were stimulated *with C*. *muridarum* for 0, 1 and 2 hours after infection. The experiments were repeated three times.

The expression of ASC, NLRP3, AIM2, CARD12, procaspase-1, and caspase-11, in unstimulated and *Chlamydia* stimulated WT and ASC^-/-^ DCs were assessed by immunoblotting (Fig 2). The results showed that ASC expression was completely abolished in ASC^-/-^ DCs. AIM2, IL-18, Pro-caspase-1 and Caspase 11 were upregulated, while NLRP3 and CARD12 appeared down regulated in ASC^-/-^ DCs compared WT DCs. There were no significant changes in the expression of caspase-8. These findings reveal that ASC has some effect on inflammasome dependent and independent pathways.

### ASC deficiency induces changes in DCs maturation and cytokine profile

**Fig 3:**
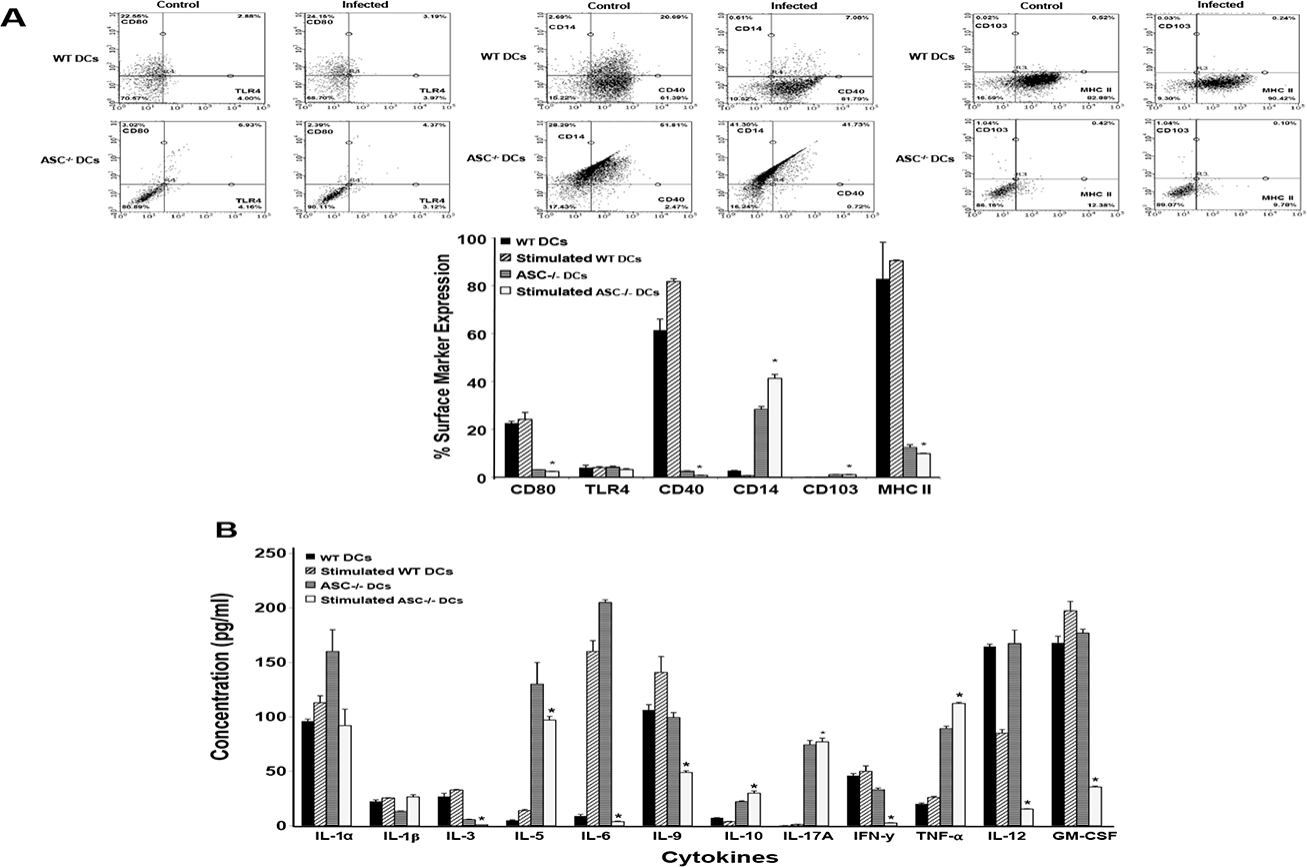
Deficiency of ASC induces changes in DCs maturation and cytokine profile. A). WT and ASC^-/-^ DCs stimulated at a ratio of 1:5 with *C*. *muridarum* for 2 hours were stained with antibodies against maturation and activation markers (CD80, CD103, MHC II, CD40, CD14, TLR4, and CD11c) conjugated with mAbs to FITC, APC and PE. The cells 10,000 events were analyzed by flow cytometry. For each sample, at least 100,000 events were collected. Data was analyzed with GuavaSoft 2.7. B). Culture supernatants from WT and ASC^-/-^ DCs stimulated at a ratio of 1:5 with *C*. *muridarum* for 2 hours were collected and cytokines were analyzed using a Luminex machine. Cytokine concentration was extrapolated from a standard calibration curve. The mean and SD of all replicate cultures were calculated. Experiments were repeated two times. *, P<0.05.

To determine if ASC deficiency regulate DCs activation and function, we measured the expression of activation markers and the cytokine profile of *Chlamydia* stimulated WT and ASC^-/-^ DCs. The results showed that after 2 hours of stimulation, the expression of the DC activation and maturation surface markers CD80 (WT 24.15% vs ASC^-/-^ 2.39%), CD40 (WT 81.79% vs ASC^-/-^ 0.72%), and MHC II (WT 90.42% vs ASC^-/-^ 9.78%) were significantly downregulated in ASC^-/-^ DCs whereas, CD 103 (WT 0.03% vs ASC^-/-^ 1.04%) and CD14 (WT 0.61% vs ASC^-/-^ 41.30%) were significantly upregulated. There was no significant difference in TLR4 expression in WT (3.97%) compared to ASC^-/-^ (3.12%) (Fig 3A). Also, ASC^-/-^ DCs exhibited a different cytokine profile compared to WT DCs. The cytokine profile showed that there was a significant decrease in the Th1 cytokines; IL-3, -6, -9, -12 (70), GM-CSF, and IFN-ϒ and a significant increase in the Th2 cytokines; IL-5, -10 and -17A in infected ASC^-/-^ DCs (Fig 3B). Interestingly, IL-1α and IL-1-β expression were not significantly different between stimulated WT and ASC^-/-^ DCs, suggesting that ASC^-/-^ DCs secrete IL-1-β via an alternative pathway. TNF-α levels was statistically higher in infected ASC^-/-^ DCs, which may indicate a potential risk of immunity-induced tissue damage.

### ASC regulates DCs antigen processing events in *vitro* and in *vivo*

**Fig 4:**
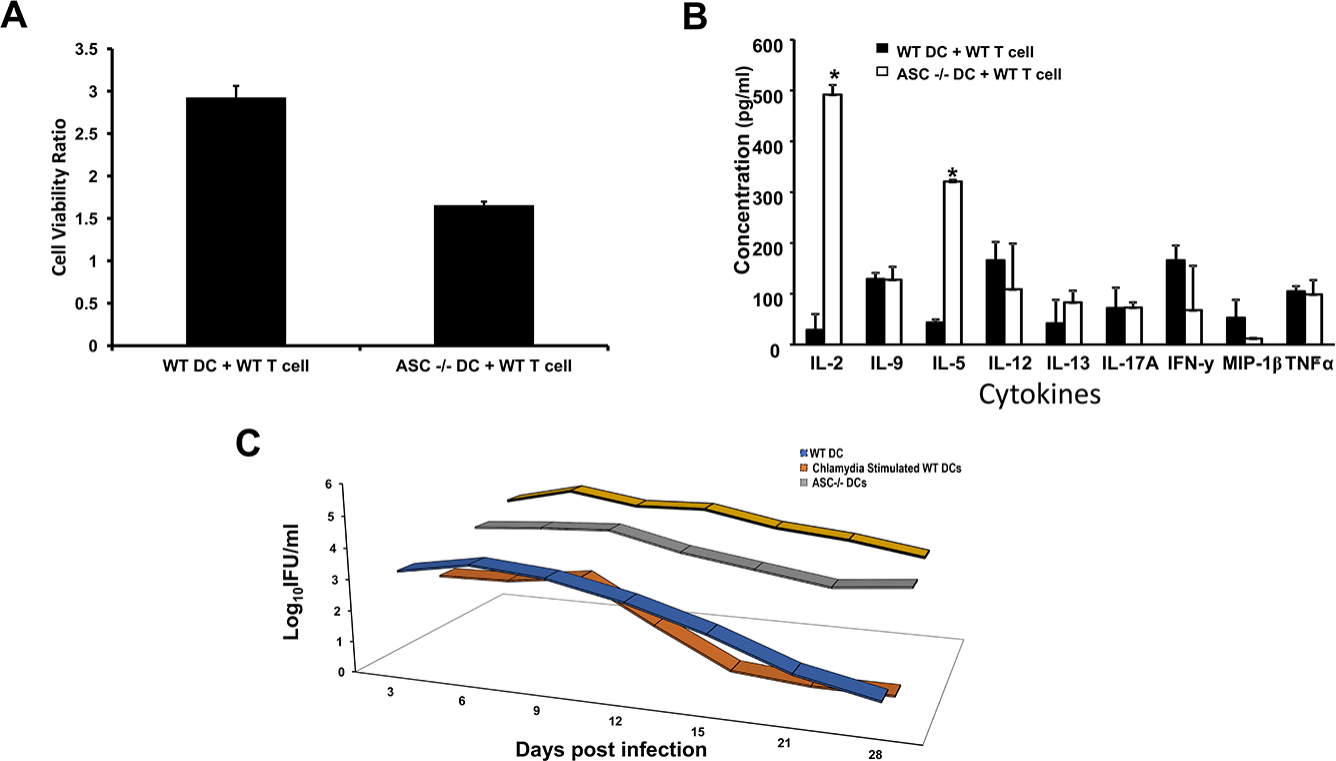
ASC regulates DCs antigen processing events *in vitro* and i*n vivo*. WT and ASC^-/-^ DCs were γ-irradiated and stimulated with UV-inactivated *C*. *muridarum* and then co-cultured with splenic T cells from immune animals. A). T cell proliferation was determined using Cell Signaling XTT Cell Viability Kit protocol. Results are expressed as cell viability ratio, which is the ratio between the absorbance values of stimulated and non-stimulated cells and the bars represent the mean and SD of three independent experiments. B). The supernatants were collected and cytokines amounts determined using a 23 plex multiplex assay from Bio-Rad. The concentration of the cytokines for each sample was obtained by extrapolation from a standard calibration curve generated simultaneously. Data was calculated as the mean and SD for triplicate cultures in each experiment. The results were from 2 independent experiments and are reported as mean cytokine concentrations (pg/ml) ± SD. Results were compared using Student’s t-test. *, P<0.05. C). Isolated DCs from WT and ASC^-/-^ mice were stimulated with *C*. *muridarum* and adoptively transferred into naïve WT mice. Mice were then infected intravaginally with live *C*. *muridarum* one week after the adoptive transfer. Infection was monitored by periodic cervicovaginal swabbing every 3 days for 2 weeks and then once every week. The *C*. *muridarum* IFUs was determined using standard methods with anti-chlamydial antibodies from Bio-Rad. The experiment was repeated three times.

Since DC activation was impaired by ASC deficiency we decided to evaluate the effect of ASC deficiency on the DC antigen presenting function by measuring *Chlamydia*-induced T cell proliferation and T cell cytokine profile *in vitro*. In addition, we determined the infectivity of WT mice that had been adoptively transferred with WT and ASC^-/-^ DCs respectively. Data showed a significant impairment in the ability of ASC^-/-^ DCs to induce Ag-specific proliferation of T cells (p< 0.012) (Fig 4A). Cytokine profile revealed a decrease in IL-12, IFN-y, and MIP-1β in the media of T cells co-cultured with ASC^-/-^ DCs, while IL-2 and -5 were significantly increased, compared to T cells co-cultured with WT DCs (Fig 4B). The increase in IL-2 might have anti-immune enhancing function, such as promoting activation-induced cell death of T cells and downregulating antigen specific T cell numbers after the clonal expansion phase of an immune response [34]. This suggests that ASC deficiency in DCs diminishes their capacity to activate elevated T cell response and secrete Th1 cytokines against *Chlamydia* infection. To provide further evidence to support our hypothesis, the course of infection in adoptively transferred mice was monitored (Fig 4C). Following infection, mice that received *Chlamydia* stimulated WT DCs showed a remarkably reduced infection burden (~2-log less IFUs) and resolved the infection earlier (15 days post infection). Whereas, mice adoptively transferred with *Chlamydia* stimulated ASC^-/-^ DCs had high IFUs till day 28. The adoptive transfer data indicates that the loss of ASC in DCs impairs anti-chlamydial immunity in the host thus leading to high bacterial burden and the longer duration it takes to clear the chlamydial infection.

### Effect of ASC on DCs immunometabolism

**Fig 5:**
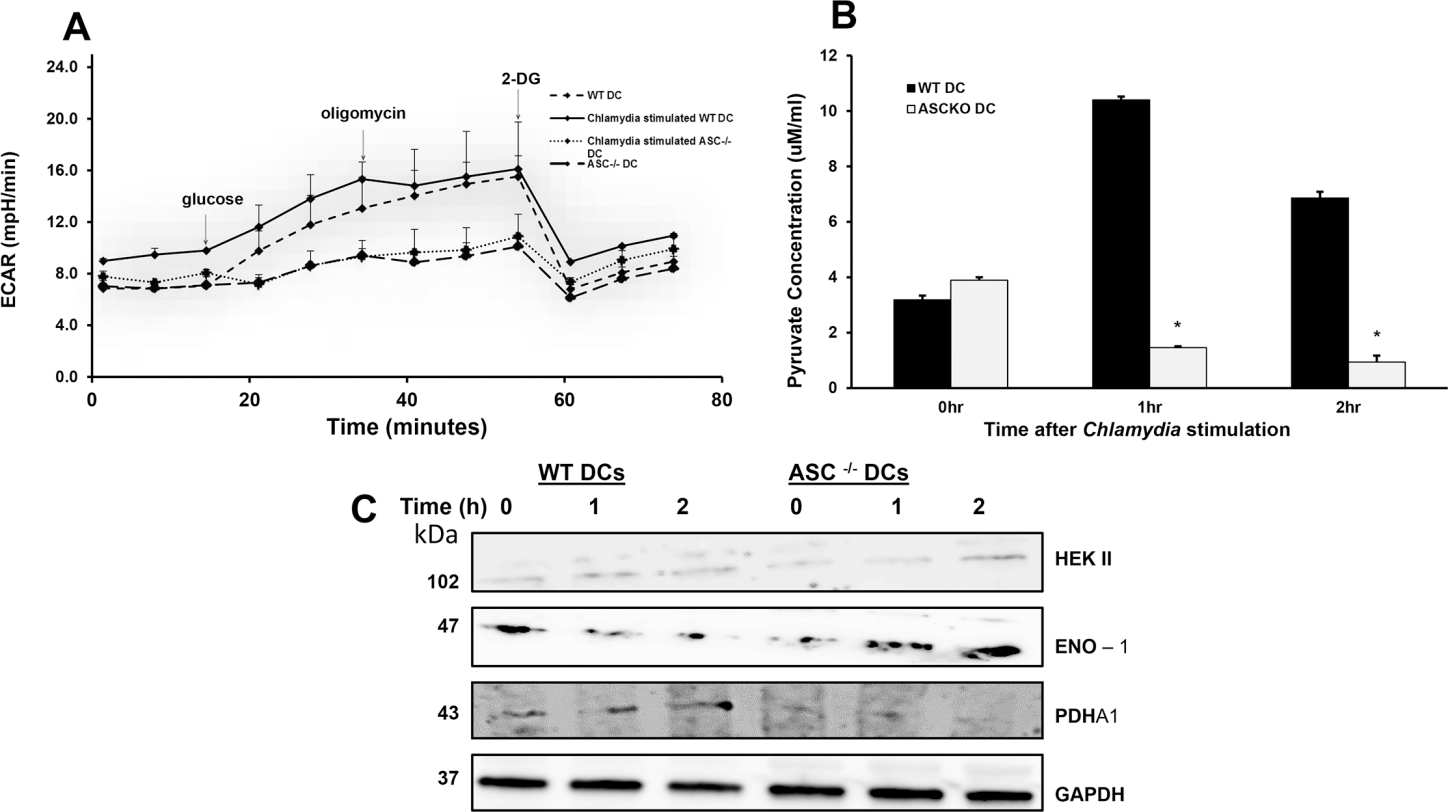
Effect of ASC on DCs glycolytic pathway. To determine the influence of ASC on DC glycolysis DCs. A). DCs were stimulated with *C*. *muridarum* for 30 minutes and real-time ECAR was measured in a live DCs using an XFe-96 analyzer. Vertical lines indicate addition of glucose (glycolysis substrate), oligomycin (ATP synthase inhibitor), and 2-DG (glycolysis inhibitor). Graphs in this figure represent the mean ± SD of three independent experiments. B). Culture supernatants from WT and ASC^-/-^ DCs stimulated at a ratio of 1:5 with *C*. *muridarum* at 0, 1 and 2 hours were collected, and their pyruvate concentration in μM/ml was determined. Data were calculated as the mean ± SD for triplicate cultures from each experiment (*, P < 0.05). Control groups included uninfected DCs and culture media. n = 12 pooled from three independent experiments. In ASC^-/-^ DCs, the pyruvate production was markedly reduced compared to WT DCs after *Chlamydia* stimulation. C). Western blotting was used to analyze HEK II, ENO-1 and PDH in lysates from *C*. *muridarum* stimulated WT and ASC^-/-^ BMDCs after 0, 1 and 2 hours. The expression levels of the glycolytic enzymes HEK II and ENO-1 in WT and ASC^-/-^ DCs were unchanged before or after *C*. *muridarum* stimulation. PDH expression was downregulated during *C*. *muridarum* stimulation of ASC^-/-^ DCs.

The metabolism, specifically glycolysis, plays a critical previously unrecognized role in DCs anti-microbial response. Therefore, we investigated the impact of ASC on the glycolytic pathway in DCs. We hypothesized that ASC deficiency might bring about changes in the DCs metabolic pathway. To determine the effect of ASC deficiency on the metabolism of *Chlamydia* stimulated DCs, we evaluated DC glycolytic function (measured by extracellular acidification rate, ECAR, as an indication of aerobic glycolysis in live cells), pyruvate production and the expression of glycolytic enzymes. Our results showed in *Chlamydia*-exposed WT DCs ECAR was elevated upon addition of glucose and persisted after treatment with oligomycin, an ATP synthase inhibitor added to induce maximal glycolysis. Whereas the ECAR change in *Chlamydia* stimulated ASC^-/-^ DCs was minimal upon the addition of glucose (Fig 5A). The absence of an increase in glycolytic rate in *Chlamydia*-treated ASC^-/-^DCs was confirmed by determining the production of pyruvate. ASC^-/-^ DCs were shown to have markedly reduced Pyruvate production compared to WT DCs after stimulation with *Chlamydia* (P<0.001) (Fig 5B). There was no differential expression of the glycolytic enzymes hexokinase II (HEK II), enolase-1 (ENO-1), and triose phosphate isomerase (TIM) in WT and ASC^-/-^ DCs before or after *Chlamydia* stimulation (Fig 5C). Interestingly, Pyruvate dehydrogenase (PDH) expression decreased with time in *Chlamydia* stimulated ASC^-/-^ DCs. PDH acts as a link between glycolysis and the tricarboxylic acid (TCA) cycle, which plays a role in protein acetylation and fatty acid synthesis. This result suggests that in the absence of ASC, PDH production is inhibited thus leading to a decrease in the needed interchange in metabolism of DCs with the downregulation in the conversion of pyruvate into acetyl-coA. This enzyme along with the metabolites it produces is required to meet the increased bioenergetics and biosynthetic demands of an activated DC, specifically by funneling metabolites into pathways for lipid and protein synthesis. Our data indicates that DC activation and function in response to *Chlamydia* stimulation was accompanied by the increased in aerobic glycolytic rate and pyruvate production, which in the absence of ASC was decreased.

**Fig 6:**
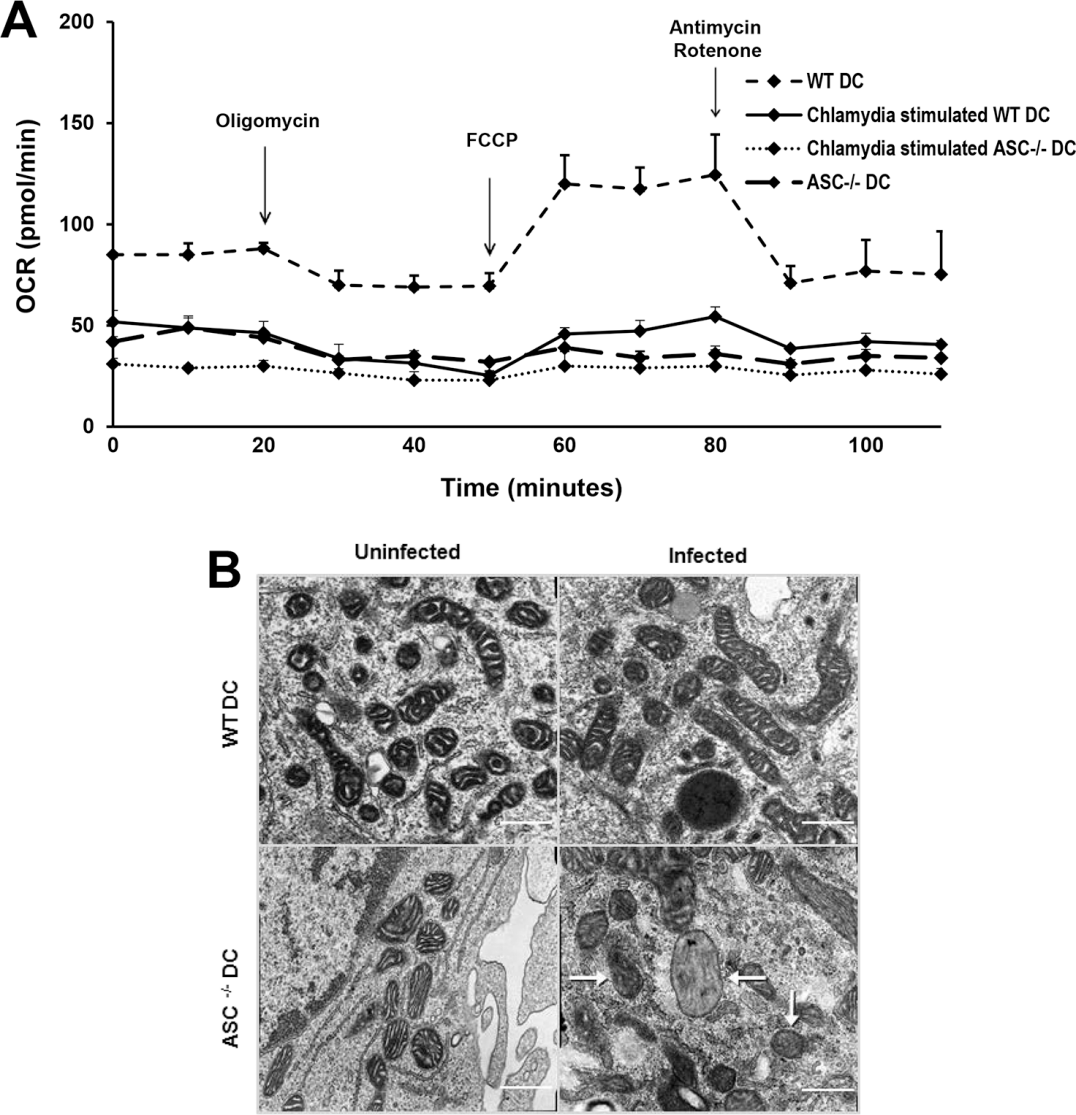
Effect of ASC on DCs mitochondrial respiration and morphology. We analyzed the effect of *Chlamydia* on WT and ASC^-/-^ DCs mitochondrial respiration and morphology using XF extracellular flux analyzer assay and electron transmission microscopy (TEM) respectively. A). WT and ASC^-/-^DCs were stimulated with *C*. *muridarum*. After 24 h, mitochondrial function was assessed. Real-time changes in oxygen consumption rates (OCRs) in a live cell were analyzed using extracellular flux analysis. Where indicated, OCRs were analyzed in response to 1 μM oligomycin, 1.5 μM fluoro-carbonyl cyanide phenylhydrazone (an uncoupling agent that disrupts ATP synthesis by transporting hydrogen ions through a cell membrane before they can be used to provide the energy for oxidative phosphorylation), and 100 nM rotenone plus 1 μM antimycin A that in combination shuts down mitochondrial respiration and enables the calculation of non-mitochondrial respiration driven by processes outside the mitochondria (all from Sigma-Aldrich), as indicated. Graphs in this figure represent mean values ± SD of three independent experiments. B). The WT and ASC^-/-^ DCs were infected with *C*. *muridarum* at a ratio of 1:5 for 2 hours. A JEOL 1200EX transmission electron microscope was used to examine mitochondrial changes in the fixed DC slices. Magnification used was 30,000X; scale bar indicates 400nm. The ultrastructure of the tubular mitochondria was well defined in unstimulated WT, ASC^-/-^ and *C*. *muridarum* stimulated WT DCs. *C*. *muridarum* stimulated ASC^-/-^ DCs did not have well-defined mitochondrial morphology and their cristae appear degraded. The experiment was repeated three times.

Changes in glycolytic rate, pyruvate production and PDH expression in *Chlamydia* stimulated ASC^-/-^ DCs prompted us to analyze DC mitochondrial oxygen consumption rate (OCR) and morphology to determine whether ASC deficiency is critical for mitochondria respiration. A detailed analysis showed OCR changes WT DCs in response to oligomycin and antimycin-A/rotenone. *Chlamydia* stimulated DCs showed that there was a shift in metabolism towards glycolysis to obtain quick ATPs to meet DC activation needs. However, *Chlamydia* stimulated ASC^-/-^ DCs were entirely nonresponsive to these drugs (Fig 6A). The mitochondria ultrastructure was well preserved in unstimulated WT and ASC^-/-^ DCs and there were no visible alterations in *Chlamydia* stimulated WT DCs. However, in *Chlamydia* stimulated ASC^-/-^ DCs we observed some discernible changes in the mitochondrial morphology, showing that they have undergone a gradual cristae disruption with few cristae remaining (Fig 6B). These findings suggest that ASC deficiency is associated with mitochondrial stress and cristae dynamics.

## Discussion

In this study, we have shown that ASC regulates DC maturation and function during *Chlamydia* infection by changing the expression of some inflammasome associated proteins and altering core DC metabolism through glycolysis and mitochondrial regulation. Our results showed that AIM2, IL-18, Caspase-1 and Caspase 11 were upregulated, while NLRP3 and CARD12 appeared down regulated. The down regulated proteins NLRP3 and CARD12 form part of ASC dependent inflammasomes, hence their lower expression in ASC^-/-^ DCs was predicted. However, we observed an increased and constitutive expression of AIM2 in *Chlamydia* stimulated ASC^-/-^ DCs, even though it is also another inflammasome protein associated with ASC. This corroborates another study that has reported that AIM2 protein acts independently of the inflammasome and IL-1β in colitis-associated colon cancer [35]. AIM2 increase appears to be associated with increased pathology and immunosuppressive DCs. AIM2 has been shown to promote calcium efflux and reactive oxygen species from mitochondria, leading to calpain activation and high levels of IL-1α, which facilitates immune suppression. Human immunosuppressive DCs produce higher levels of IL-1α than healthy pDCs in an AIM2-dependent manner [36, 37]. The high expression of AIM2 and IL-1α demonstrates that ASC^-/-^ DCs may lead to increased female genital pathology. The increased expression of Caspase-11 we noticed is interesting since it is a non-canonical pathway involved in the production of IL-1β and IL-18 which is necessary for innate immune response and pyroptosis [38–40]. Thus, the increased expression of Caspase- 11, is a compensatory mechanism by the ASC^-/-^ cells to have a functioning inflammasome in the absence of the other inflammasome assemblies. The expression of Caspase-1 and IL-18 that we see in the ASC^-/-^ DCs can be attributed to the expression of caspase-11.

Our data shows a link between ASC and the regulation of DC maturation and function. The DC maturation markers CD80, CD40, and MHC II were significantly downregulated in ASC^-/-^ DCs compared to WT DCs while, CD103 and CD14 were upregulated. CD103^+^ DCs are the subject of interest due to their role in regulating mucosal immunity, the induction of tolerogenic immune responses and imprinting mucosal homing phenotypic changes on antigen-specific T cells [41–43]. CD14 is a fascinating molecule with a wide range of functions and associations, and has been shown to respond to bacteria LPS through its interaction with toll like receptors [44], and increase in CD14 in monocytes have been associated with increased disease in HIV infected individuals [45]. In addition, the rapid accumulation of CD14^+^ CD11c^+^ DCs has been shown to precede changes in gut mucosal architecture, indicating that DCs with these receptors may be directly involved in disease immunopathology [46, 47]. IL-1β activated dendritic cells can use CD14 mediated mechanisms to stimulate T cells into producing IL-17 [48]. Thus, the increase in these surface markers on DCs might be associated with the increased infectivity that we have observed in ASC^-/-^ mice. Also, we noticed a significant decrease in Th1 cytokine secretions (IL-3, -9, -12, GM-CSF, IFN-ϒ) and an increase in Th2 cytokines (IL-5, -10 and -17A) in ASC^-/-^ DCs compared to WT DCs, suggesting that in contrast with *Chlamydia* infected WT DCs, *Chlamydia* infected ASC^-/-^ DCs delayed maturation and promoted Th2-mediated immune response, which does not provide efficient protection against genital *Chlamydia* infection [7, 49]. The IL-18 might be playing a role in tamping down the effect of the elevated IL-5 and IL-6 after stimulation with Chlamydia. The postponement of DCs maturation is an important factor that is associated with the impairment of T cell activation. Early maturation of DCs leads to the rapid acquisition of capacity to express crucial costimulatory molecules such as IL-1β, CD40, and IL-12 that are important for the activation of differentiation of T cells towards the Th1 phenotype.

*Chlamydia* infected ASC^-/-^ DCs had delayed maturation and promoted Th2-mediated immune response, so we evaluated the effect of ASC deficiency on the DC antigen presenting function by measuring *Chlamydia*-induced T cell proliferation and T cell cytokine profile *in vitro*. Data from the experiment showed that ASC^-/-^ DCs ability to induce Ag-specific proliferation of T cells was impaired. This makes sense since we had shown that these cells were not fully matured and mediated Th2 response. However, results from the cytokine profile showed a significant increase in IL-2 and -5 in the media of T cells co-cultured with ASC^-/-^ DCs compared with T cells co-cultured with WT DCs. IL-2 is known as an enhancer of lymphocyte proliferation, however, IL-2 has been reported to play another role other than T cell proliferation, which is involves self-tolerance, which might lead to the down regulation of T cell response and eventually cause activation-induced cell death (AICD) in T cells [34]. Our results thus imply that the IL-2 produced by T-cells in the presence of ASC^-/-^ DCs might not necessarily be assisting in the proliferation of these T cells but in fact be responsible for the reduced proliferation that we observed in these T cells. The regulation of T cell susceptibility to AICD is governed by the stage of T cell maturity, the previous activation history of the T cell, and the presence or absence of antigen-presenting cells (APC) [50]. We have shown that the ASC^-/-^ DCs were not mature, and this might play a role in the T cells not being able to become fully matured thus making them susceptible to AICD. It was observed that previous exposure of both CD4^+^ and CD8^+^ T cells to IL-2 increases susceptibility to AICD [51]. This discrepancy is due to differences in the timing of exposure to IL-2, with the increased susceptibility to T cells previously exposed to IL-2. This may be due to IL-2-induced up-regulation of FasL expression and the suppression of expression of FLICE-inhibitory protein (FLIP), an inhibitor of Fas signaling [52, 53]. It might also be because of the timing of the production of the IL-2 in the T cells co-cultured with ASC^-/-^ DCs was not appropriate. In addition to the significant increase in IL-5 would lead to increased Th2 response which is not protective during chlamydia infection. This suggests that ASC deficiency in DCs diminishes their capacity to activate elevated T cell response and secrete Th1 cytokines against *Chlamydia* infection.

To confirm our *in vitro* data, we extracted DCs from ASC^-/-^ mice and exposed the DCs to *Chlamydia*, and then adoptively transferred the DCs to naive WT mice. The mice were infected with *Chlamydia* after adoptive transfer and we then monitored the course of infection. The mice that were adoptively transferred with ASC^-/-^ DCs did not clear their *Chlamydia* infection compared with mice that were adoptively transferred with WT DCs. *In vitro* and *in vivo* results prove that ASC deficiency negatively regulates DC maturation and APC function.

To further investigate the mechanism by which ASC deficiency impairs DCs function, we evaluated the effect of ASC on DCs metabolism after *Chlamydia* stimulation. Living cells, such as DCs, use either mitochondrial respiration and/or glycolysis to meet metabolic. ECAR and OCR provides insight into glycolytic activity and mitochondrial function, by measuring cellular metabolism in live cells in real time. During the process of DCs maturation there is a shift toward the glycolytic metabolic state from OXPHOS. However, tolerogenic dendritic cells favor OXPHOS and fatty acid oxidation [29] [30]. Our ECAR data indicated that WT DCs, after exposure to *Chlamydia*, began reprograming metabolism towards glycolysis promoting rapid DC maturation and action to induce quick activation of T cells differentiation towards the Th1 phenotype. In absence of ASC, DCs did not respond to glucose, which the cells utilize through the glycolytic pathway to produce ATP. In addition, the pyruvate concentration in supernatants from ASC^-/-^ DCs was significantly reduced compared to that of WT DCs when stimulated with *Chlamydia*. In DCs, increase in glycolysis supports lipid biosynthesis by facilitating the expansion of the endoplasmic reticulum and Golgi apparatus and increasing the biosynthetic capacity that is essential for the function of mature DCs. In the absence of ASC, DCs glycolytic pathway was rapidly impaired resulting in decreased flux of carbon molecules from the glycolytic pathway into the tricarboxylic acid (TCA) cycle, which is essential for meeting the demands of fatty acid synthesis. This lack of metabolic shift in ASC^-/-^ DCs is accompanied by delayed maturation and damaged APC function. To further understand the molecular basis of ASC role on DCs cellular respiration, we investigated expression levels of the glycolytic enzymes; HEK II, ENO-1, TIM, and PDH. The results showed that ASC did not have an impact on HEK II, ENO-1 and TIM expression during *Chlamydia* stimulation. However, PDH expression was gradually reduced in *Chlamydia* stimulated ASC^-/-^ DCs. PDH catalyzes the irreversible oxidative decarboxylation of pyruvate to generate Acetyl-CoA, which is then oxidized by the TCA cycle to generate NADH and FADH2, which are used by the mitochondrial electron transport chain and ATP synthase to generate cellular energy [54–56]. The result suggests that downregulation of PDH in ASC^-/-^ DCs causes a deficit in energy production and fatty acid biosynthesis which impairs DC differentiation and function.

The marked difference in ECAR in *Chlamydia* stimulated ASC^-/-^ DCs prompted us to study mitochondrial function and morphology, which are important in innate immune response and have indeed become critical regulators of DC responses against pathogen infection [31]. Mitochondrial stress test and TEM were used to compare OCR and DCs ultra-structure. The OCR data indicated that the mitochondria of *Chlamydia* stimulated WT DCs had reduced capacity to produce ATP, which is in line with the ECAR result, suggesting that *Chlamydia*-mediated activation of DCs is associated with a metabolic transition in which mitochondrial oxidative phosphorylation is inhibited and the cells become committed to glucose and aerobic glycolysis for survival. However, in *Chlamydia* stimulated ASC^-/-^ DCs the mitochondria have lost respiratory function and showed visible damage in the mitochondrial cristae. ASC is localized within the mitochondria and recruits Bax to the membrane of mitochondria [57]. Cristae are folds of the inner mitochondrial membrane and allow the production of ATP through the electron transport chain and chemiosmosis. It is now clear that mitochondrial cristae are dynamic bioenergetic compartments whose shape changes under different physiological conditions [58]. There appears to be a connection between cristae shape and OXPHOS function that has an impact on cellular metabolism.

In this study, we report that the changes in the metabolism of DC induced by *Chlamydia* are critical for establishing DC immune cell phenotype and are necessary for immune response against *Chlamydia* infection. We also associate ASC with metabolic reprogramming of DC after exposure to *Chlamydia*. Our study demonstrates a critical role for ASC in DCs metabolism, which regulates DC response to Chlamydia infection. Development of strategies that will enhance DC metabolic pathways potentially improving antibacterial responses, represents one possible application of this discovery. However, we are only beginning to understand the extent to which ASC regulation of metabolism is interlinked with the functional properties of DCs. There are still questions to be asked about the molecular basis and reaction site of ASC that has a direct impact on DC metabolism, and there are likely to be numerous opportunities for novel therapeutic strategies that modulate this ASC-metabolic regulatory axis.
